# Supplementary material for: Ni-Catalyzed Enantioselective Intramolecular Mizoroki–Heck Reaction for the Synthesis of Phenanthridinone Derivatives
Source: J Org Chem. 2023 Jun 15;88(13):8203–26. doi: 10.1021/acs.joc.3c00202 (PMC10337041; doi:10.1021/acs.joc.3c00202)
Supplement: Supplementary file 2 — jo3c00202_si_002.zip [file jo3c00202_si_002.zip › Deprotected Heck products/3e iPr-NH/1H iPr-NH 3e/2/pdata/1/email_DR-107-129 pure_2_1.pdf]

DR-107-129 pure COSY
COSYGPSW CDCl3 {D:\nmrusers\malachowski} BM 7

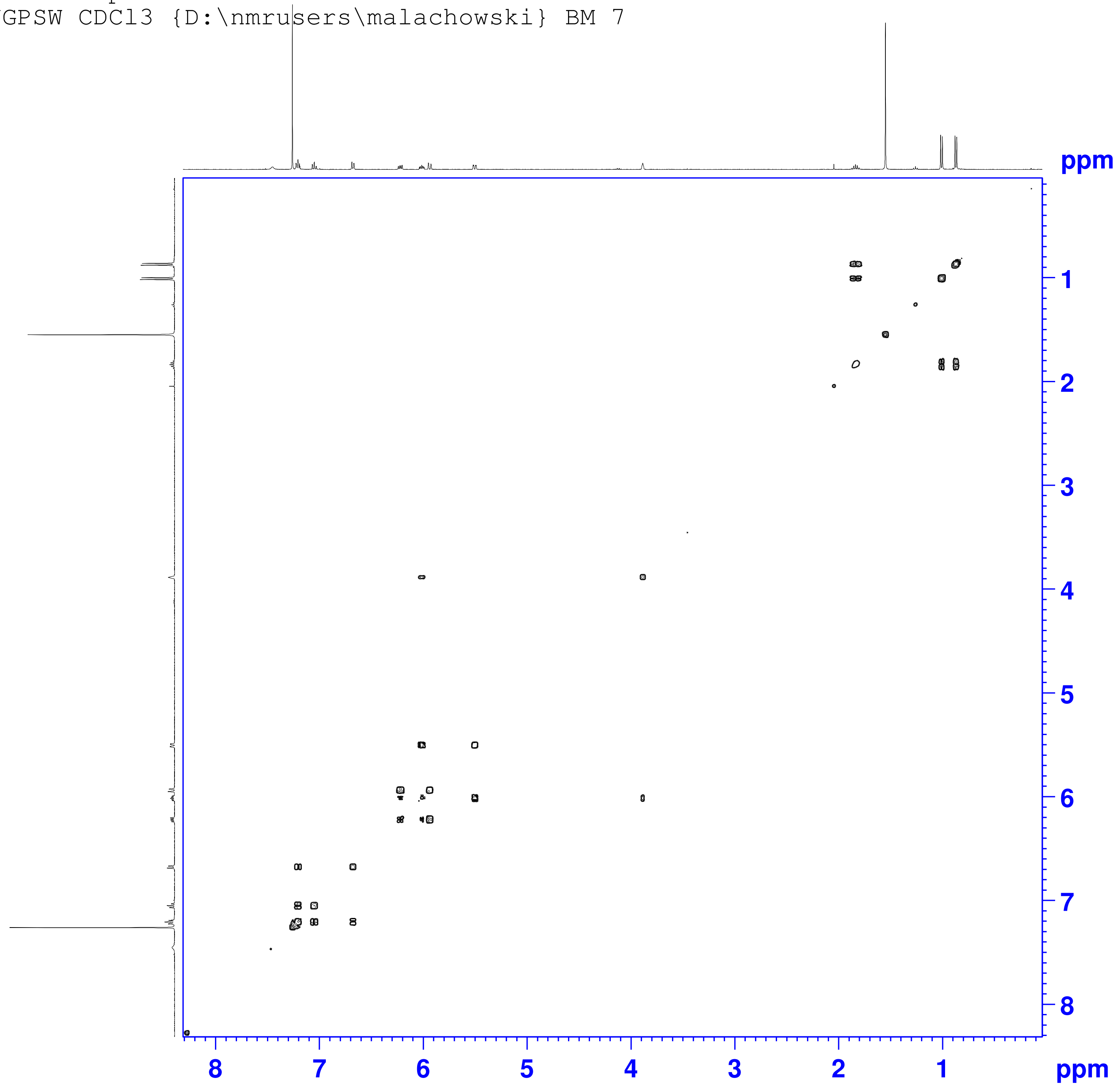

BRUKER

Current Data Parameters	
NAME	DR-107-129 pure
EXPNO	2
PROCNO	1
F2 - Acquisition Parameters	
Date_	20210401
Time	13.09 h
INSTRUM	spect
PROBHD	Z104450_0352 (
PULPROG	cosygpppqf
TD	2048
SOLVENT	CDCl3
NS	1
DS	16
SWH	3311.258 Hz
FIDRES	3.233651 Hz
AQ	0.3092480 sec
RG	203
DW	151.000 usec
DE	6.50 usec
TE	298.0 K
D0	0.00000300 sec
D1	1.88777006 sec
D11	0.03000000 sec
D12	0.00002000 sec
D13	0.00000400 sec
D16	0.00020000 sec
IN0	0.00030200 sec
TDav	1
SFO1	400.1516808 MHz
NUC1	1H
P0	12.75 usec
P1	12.75 usec
P17	2500.00 usec
PLW1	12.14200020 W
PLW10	2.19309998 W
GPNAM[1]	SMSQ10.100
GPZ1	10.00 %
P16	1000.00 usec
F1 - Acquisition parameters	
TD	128
SFO1	400.1517 MHz
FIDRES	51.738411 Hz
SW	8.275 ppm
FnMODE	QF
F2 - Processing parameters	
SI	1024
SF	400.1500095 MHz
WDW	QSINE
SSB	0
LB	0 Hz
GB	0
PC	1.40
F1 - Processing parameters	
SI	1024
MC2	QF
SF	400.1500095 MHz
WDW	QSINE
SSB	0
LB	0 Hz
GB	0
